# The Antimicrobial and Healing Effect of *Scrophularia striata* Boiss Hydroalcoholic Extract on First- and Second-Grade Pressure Wounds in Patients with Brain and Spinal Cord Injury: A Randomized Clinical Trial

**DOI:** 10.1155/2022/8522937

**Published:** 2022-11-28

**Authors:** Zahra Bagheri, Amir Larki-Harchegani, Shabnam Pourmoslemi, Amir Nili-Ahmadabadi, Ebrahim Bakhtiari, Hamid Safarpour, Ali Fathi Jouzdani, Morteza Shamsizadeh

**Affiliations:** ^1^School of Nursing and Midwifery, Student Research Committee, Hamadan University of Medical Sciences, Hamadan, Iran; ^2^Department of Pharmacology Toxicology, School of Pharmacy Hamadan University of Medical Science, Hamadan, Iran; ^3^Department of Pharmaceutics, Medicinal Plants and Natural Products Research Center, School of Pharmacy, Hamadan University of Medical Sciences, Hamadan, Iran; ^4^Isfahan University of Medical Science, Isfahan, Iran; ^5^Department of Health in Disasters and Emergencies, School of Public Health and Safety, Shahid Beheshti University of Medical Sciences, Tehran, Iran; ^6^Neuroscience and Artificial Intelligence Research Group (NAIRG), Student Research Committee, Hamadan University of Medical Sciences, Hamadan, Iran; ^7^USERN Office, Hamadan University of Medical Sciences, Hamadan, Iran; ^8^Chronic Diseases (Home Care) Research Center, Hamadan University of Medical Sciences, Hamadan, Iran

## Abstract

**Materials and Methods:**

This double-blind clinical trial study was performed on grade 1 and 2 pressure wounds in 120 patients with cerebral-spinal cord lesions. The patients were randomly divided into four groups (*n* = 30). Topical treatments in all groups were performed twice a day. These groups included experiment 1 (SHE + phenytoin), experiment 2 (SHE + SHE), control (phenytoin + phenytoin), and placebo (eucerin + phenytoin). After evaluating the effect of SHE on wound healing, its antibacterial activity was determined by the standard agar well diffusion method.

**Results:**

Patients in each group in this study did not significantly differ in demographic and clinical variables. Complete wound healing by the 10^th^ day of the intervention occurred in 63%, 100%, and 27% of patients in experimental 1, experimental 2, and control groups, respectively. In contrast, the placebo group had no complete wound healing until the 10^th^ day. Topical application of SHE, twice a day in the experimental 2 groups, had a higher potency to heal wounds and reduce the duration of complete wound healing in patients compared with other groups.

**Conclusion:**

SHE, as a novel treatment option, has good potential to accelerate the healing of first- and second-degree pressure wounds in patients with brain-spinal cord injuries.

## 1. Introduction

Pressure ulcers are a common skin injury among patients with brain and spinal cord lesions and permanent movement disorders. In addition to causing pain and decreased function, this complication increases the risk of bone and blood infections, lengthens the hospital stay, and necessitates additional surgical procedures [1–3]. As a result of constant pressure placed on the skin for an extended period, pressure ulcers can destroy the skin and the underlying tissues, ultimately leading to tissue necrosis and loss of nutrition and oxygen [4]. Patients in long-term care facilities, rehabilitation centers, and intensive care units (ICUs) are susceptible to pressure ulcers at up to 23% [5]. According to the Agency for Healthcare Research and Quality (AHRQ), over 2.5 million Americans suffer from pressure ulcers, and 60,000 people die of pressure ulcers each year [5]. Medical management of pressure ulcers costs the US healthcare system between $9.1 billion and $11.6 billion annually because of increased healthcare utilization [5]. The prevalence of pressure ulcers in European hospital inpatients is above 18%, with only 10% of patients receiving appropriate preventive care [6]. In Iran, the prevalence of pressure ulcers in hospital patients is 44% [7, 8]. Consequently, preventing and treating pressure ulcers can reduce suffering, long-term hospitalisation, patient mortality, and healthcare costs [7].

There are many sites where pressure ulcers can form, including the tibia, scapula, scapular, lumbar spine, iliac crest, sacrum, elbows, heels, and ankles [9]. The techniques include changing the patients' conditions, not scrubbing the wound, applying dressings, lubricants, alginates, or supportive dressings to protect the wound from friction, nutritional support, and physical therapy measures, and rehabilitation to assist patients in walking again [10, 11]. Furthermore, traditional medicine has been considered in studies today as a means of preventing and treating pressure ulcers due to the side effects of chemical drugs. Therefore, using wax, honey, some plant extracts, and oils such as those from sunflower, olive, and borage roots is recommended for treating pressure ulcers [8, 12]. The use of natural antibiotics, such as drugs and antibiotics derived from plants, is regarded as efficacious [13]. In the western part of Iran, the Zagros Mountains are home to one of the most important medicinal plants, *Scrophularia striata*, also known as figwort. In Ilam province (West Iran), this plant species has been used for treating many different illnesses for many years, despite not knowing its chemical composition [14, 15]. In this sense, *Scrophularia striata* is a plant species that heal wounds and prevents bacterial infections [16, 17].

The genus *Scrophularia* includes 300 species of *Scrophularia striata* [18]. Plant extracts of this plant contain flavonoids, monoterpenes, and coumarins, which are antioxidants and antibacterials. *Scrophularia striata* reduce edema, cell infiltration, and proliferation of T lymphocytes by inhibiting the production of prostaglandin E2, which is responsible for producing interferon-gamma and tumour necrosis factors [18, 19]. Additionally, phenolic acids with antibacterial properties are found in this plant, contributing to its effectiveness as a wound healer [20]. It has been demonstrated that *Scrophularia striata* have properties that stimulate fibroblast regeneration, antioxidant activity, and anti-inflammatory activity and promote faster wound healing, angiogenesis, and vasodilation [21]. In samples treated with *Scrophularia striata* extract, an increase in fibroblast cells and collagen fibres with the formation of epidermis and dermis has been observed at the wound site [22].

Since pressure ulcers are considered a life-threatening condition for patients with movement disorders caused by brain and spinal cord lesions, using various treatment and care methods, especially herbal medicines, is vital to quickly and completely prevent and treat pressure ulcers. In lab experiments, *Scrophularia striata* have shown properties to be anti-inflammatory, antibacterial, and antioxidant. In this study, we examined the effect of hydroalcoholic *Scrophularia striata* extract on healing first- and second-grade pressure ulcers in patients with brain and spinal cord lesions.

## 2. Materials and Methods

### 2.1. Trial Design

This randomized controlled and blinded clinical trial was conducted to evaluate the antimicrobial and healing effect of *Scrophularia striata* Boiss hydroalcoholic extract on first- and second-grade pressure wounds in patients with brain and spinal cord injury. From December 2019 to October 2020, 140 survivors with brain and spinal cord lesions were admitted to the intensive unit care of Besat, Imam Hossein, Shahid Beheshti, and Sina educational-medical centers affiliated to Hamadan University of Medical Sciences and included by the available sampling method. This study complied with the Consolidated Standards of Reporting Trials (CONSORT) statement [23]. The ethics committee approved this study of Hamadan University of Medical Sciences under the code IR.UMSHA.REC.1398.747 and was registered in the Iranian clinical trial database with the code IRCT20120215009014N328 and address http://www.irct.ir. A Helsinki Declaration guideline was also followed in the conduct of the study. The patient's consent was obtained directly after they were discharged from the ICU. Informed consent covered both study participation and consented to publish the findings. In cases where the patient lacked decision-making capacity, the patient's legal guardian or healthcare proxy could consent on the patient's behalf. The group of patients with their family members or friends was called the enrolled family. Additionally, the study was conducted by the Helsinki guideline.

### 2.2. Inclusion and Exclusion Criteria

Inclusion criteria include no history of allergy to *Scrophularia striata* extract and its products, having first- and second-grade pressure ulcers in at least 1 cm from the skin, age group of 15–75 years, no history of asthma, chronic obstructive pulmonary disease (COPD), hay fever and swelling of the nasal mucosa, skin sensitivity, lack of skin lesions preventing examining the pressure ulcers, nonuse of anti-inflammatory drugs (glucocorticoids, NSAIDs) for similar symptoms or other symptoms (such as rheumatoid arthritis), and lack of concomitant participation in another clinical trial effective on treatment of pressure ulcers. Also, the patients were excluded with withdrawal from the study earlier than one week, discharge or the personal satisfaction of the patient or transfer of the patient to a center outside Hamedan, intolerance and allergy to *Scrophularia striata* extract during the study, local skin complications not allowing the use of test cream, concomitant and indirect use of anti-inflammatory drugs (glucocorticoids, NSAIDs), forgetting or not using the extract 2 or 3 times a day (once negligible), patients with diabetes mellitus with diabetic ulcers and diabetic neuropathy, and third-grade pressure ulcers.

### 2.3. Sample Size, Randomization, and Blinding

**Figure 1:**
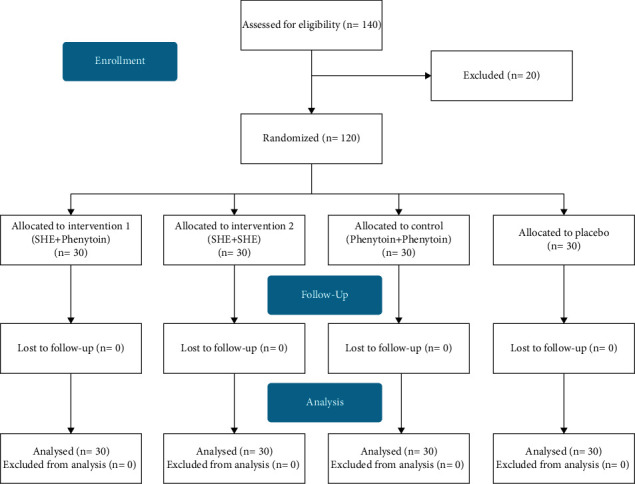
CONSORT flow diagram: in this randomized clinical trial study, 120 people were included in this study. Thirty people were allocated for each of the four study groups. SHE, *Scrophularia striata* hydroalcoholic extract.

The sample size was determined based on the study carried by Azhdarmehri et al. [28], including 120 patients. The patients were divided into four groups of 30 people considering the first type error of 0.1, the study power of 0.90, the difference in the score of pressure ulcer size up to 10 mm, and the standard deviation of 10. After selecting the patients based on the inclusion criteria and obtaining conscious written consent, the study samples were randomly divided into four groups (three experiment groups and a control group) of 30 people by quadruple random blocks (Figure 1).

### 2.4. Intervention

Experiment 1 group consisted of patients receiving 1% phenytoin topical cream every morning and 10% topical formulation of *Scrophularia striata* extract on pressure ulcers, 12 h later. In experiment group 2, the patients received 10% topical formulation of *Scrophularia striata* extract in the morning and 12 h later in the mentioned areas. In the control group, patients received 1% topical phenytoin cream every morning and 12 h later, and in the placebo group, they received 1% of phenytoin topical cream every morning and 12 h later and placebo or eucerin on the mentioned areas to the extent covering the radius of the pressure ulcers. To blind the study, the hydroalcoholic extract of *Scrophularia striata* and placebo were prepared by the research pharmacist and prepared in a tube and given to the researcher and the sample nurse (ulcers expert). The tubes containing the herbal extract were marked by *X*, and the tubes containing the placebo were marked by *Y*, and only the researcher was aware of the contents of the tubes. The examiner and nurse, the patient, and the statistical analyzer were unaware of any of the contents of the labelled tubes. To prepare the hydroalcoholic extract of *Scrophularia striata*, 1.5 kg of rhizomes and stems of this plant, after approval by the botanist in the herbarium of the Faculty of Pharmacy, were completely dried at room temperature 25°C in the shade and then formed into a dry powder by a mechanical mill. 300 g of dried plant powder was soaked in 70° ethanol solution on a shaker for 72 h until most of the active ingredients were extracted. The resulting mixture was filtered through filter paper and placed in a rotary apparatus at 50°C, 60 rpm; then, the solvent was removed and placed under the hood in a Petri dish for another week to dry and prepare the final extract. The final extract was approved by a pharmacist.

### 2.5. Data Collection

During ten days of the study, in all 4 groups, the area of the pressure ulcers was measured every 24 h until complete wound healing. All patients in the study received routine care for pressure ulcers, such as changing positions every 2 h and using a wavy mattress. This study was performed on selected patients from the hospital's onset of grade 1 and grade 2 pressure ulcer symptoms. Before intervention, skin tests were performed on patients with a topical formulation of pressure ulcers. Thus, some of this herbal formulation was tested on 5 mm of the patient's forearm skin and examined 24 h later. The intervention was stopped in case of any skin complication or allergy in the patient. Patient's demographic information, including age, gender, body mass index, occupation, education, and location, were recorded. A questionnaire was used to record the observations and diagnosis of the doctor and nurse to evaluate the extent of pressure ulcer healing during 10 days of the study. In this questionnaire, skin sensitivity to *Scrophularia striata* extract and ulcer size were recorded, measured daily and accurately at 8 am. The ulcer size was measured using transparent paper and a permanent pen, and finally, by scanning the sheets; the ulcer circumference size was interpreted by image *j* software in square millimetres. The examinations were scored daily from 1 to 4 based on pressure ulcer grades.

### 2.6. Agar Diffusion Susceptibility Tests

The susceptibility test was carried out by the standard agar well diffusion method for various concentrations of the extract against common organisms found in infectious wounds. Soybean casein digest agar plates were inoculated by spreading 100 *µ*l of *Staphylococcus aureus* and *Pseudomonas aeruginosa* suspensions at half McFarland (1.5 × 10^8^ cfu/ml) bacterial concentration [1]. 10 mm diameter wells were punched in the plates by using a sterile stainless-steel borer and filled with extract dissolved in DMSO at 10, 5, and 1 mg/ml concentrations. 10 *µ*g/ml clindamycin and ceftazidime solutions in water were used as a positive control against *Staphylococcus aureus* and *Pseudomonas aeruginosa*, respectively. After incubating the plates at 37°C for 24 h, they were evaluated by measuring the inhibition zone diameters at 0.1 mm accuracy. This test was conducted in triplicate.

### 2.7. Determination of Minimum Inhibitory Concentration (MIC) and Minimum Bactericidal Concentration (MBC)

MIC of the extract against *Staphylococcus aureus* and *Pseudomonas aeruginosa* was determined by using the macro dilution method according to the Clinical and Laboratory Standards Institute (CLSI) guidelines [2]. *Staphylococcus aureus* and *Pseudomonas aeruginosa* suspensions inoculated soybean casein digest broth medium at half McFarland bacterial concentration to a final concentration of 1.5 × 10^6^ cfu/ml. 20 mg/ml extract stock solution in DMSO was prepared. 5 ml of the extract stock solution was added to a test tube containing 5 ml inoculated broth medium to obtain a 10 mg/ml concentration. Serial dilution was continued in more than 11 test tubes to get extract solutions in the 0.003–10 mg/ml range. One test tube without adding the extract was used to show maximum bacterial growth, and another containing an uninoculated medium was used as proof for the aseptic technique. After incubation of the tubes at 37°C for 24 h, the concentration at which no growth was observed was determined as MIC [3]. Determination of MBC was performed by adding 100 *µ*l from the constituents of the wells not showing microbial growth in the MIC test on agar plates. After incubation at 37°C for 24 h, the lowest concentration of the extract that did not form any colonies was determined as MBC. These tests were conducted in three separate experiments, and quantities selected as MIC and MBC in at least two experiments were reported.

### 2.8. Statistical Analysis

Data analysis was performed by using Stata 23 software. The chi-square test was used to compare the nominal variables and the independent *t*-test. Repeated measures analysis of variance, covariance, and ANOVA were used to compare the quantitative variables. A significance level was considered 5%.

## 3. Results

In this study, a total of 140 patients were selected. Twenty patients were excluded due to early withdrawal. Finally, 120 patients' information was divided into four groups and reviewed (Figure 1). The patients in the groups were not significantly different in terms of gender, age, occupation, education, marriage, and residence (*P* > 0.05).  Table 1 shows the demographic characteristics of the patients. Body mass index in the patients of experiment group 1 was 24.60 ± 2.02, 24.13 ± 1 1.20 in experiment group 2, 23.96 ± 1.74 in the control group, and 23.97 ± 1.58 in the placebo group; this difference was not significant (*P*=0.423).

Comparing the groups in terms of the reason for hospitalisation, most patients were hospitalised in experiment group 1 (53.3%) and the placebo group (63.3%) owing to spinal cord injury (SCI). The patients in experiment group 2 (56.7%) and control (53.3%) were hospitalised mainly due to cerebrovascular accident (CVA). The hospitalisation period of most patients in all four study groups was 7–12 days after the start of the study. Most of the patients had grade 2 ulcers in all four groups. Patients in the four groups were not significantly different regarding cause, location, period of hospitalisation, and type of ulcers (*P* < 0.05).

### 3.1. Patients' Profile of Clinical Characteristics in Four Study Groups

**Figure 2:**
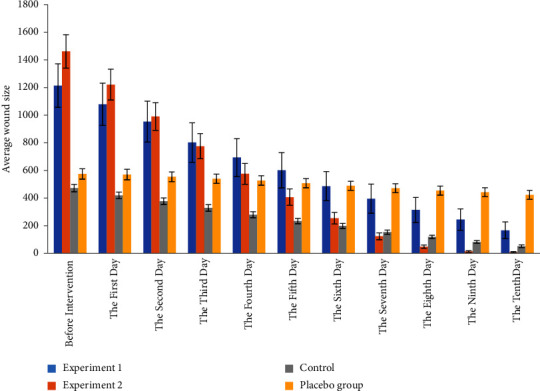
Comparison of the ulcer reduction process of participants in four study groups.

**Figure 3:**
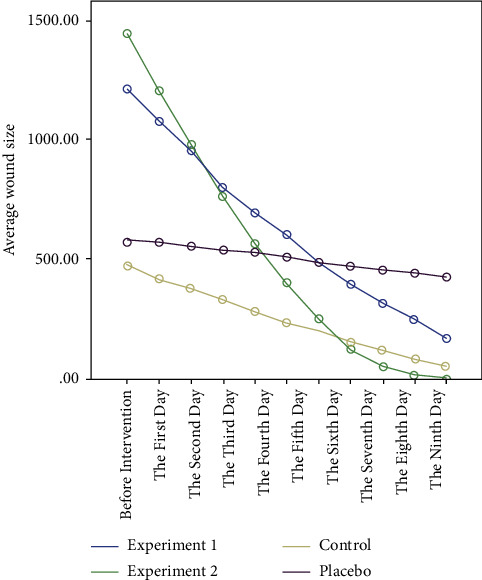
Comparison of the ulcer reduction process of research subjects in four study groups. As the diagram shows, the decreasing process slope of the mean ulcer size in patients of experiment group 2 was steeper compared with experiment group 1, control, and placebo groups.

Studying the mean ulcer size before and ten days after intervention in four groups, the results showed that there was a statistically significant difference between the mean ulcer size of four groups before intervention (*P* < 0.001) (Figure 2 and 3).  Table 2 shows patients' clinical characteristics such as the case of hospitalisation, inpatient department, hospitalisation, and wound type in four groups of study. Therefore, the four groups were not identical regarding ulcer size before intervention (Table 3). Hence, analysis of covariance showed that there was a statistically significant difference between the mean ulcer size of four groups every ten days of intervention (*P* < 0.001) (Figures 2 and 3). Furthermore, repeated measures analysis showed that in all four groups, there was a statistically significant difference between the mean ulcer size on days 1–10 (*P* < 0.001) (Figures 2 and 3). In these groups, a decreasing process in ulcer size was observed during the study days. The results of the ANOVA test and analysis of variance with repeated measures in comparison with the mean ulcer size before and after the intervention in four groups are presented in Tables 3 and 4. As the diagram shows, the decreasing process slope of the mean ulcer size in patients of experiment group 2 was steeper compared with experiment group 1, control, and placebo groups (Figure 3). According to this table, in the first to fifth days of intervention, no complete ulcer healing was observed in any of the patients. By the tenth day of intervention, 63.3% of patients in experiment group 1, 100% in experimental group 2, and 26.7% in the control group had healing ulcers. During ten days of intervention, no complete recovery was observed in any placebo patients. There was a statistically significant difference between the distribution of research subjects in terms of complete ulcers healing during ten days of intervention in three experiment group 1, experiment group 2, and the control group (*P* < 0.001), and the process of complete ulcer healing in these three groups was observed during the observation. Studying the distribution of entire ulcer healing time in four groups indicated that the time of complete ulcer healing patients in experiment group 2 was less than in group 1 and the control group (Table 5). Also, the recovery occurred on the tenth and eighth days of the intervention in 50% of patients in experiment group 1 and group 2, respectively. Based on the semiparametric log-rank test, there was a statistically significant difference between patients' complete ulcer healing time in four groups (*P* < 0.001), and the full ulcer healing time in experiment group 2 was significantly lower than the other three groups.

### 3.2. Antibacterial Activity of the Extracts against *Staphylococcus aureus* and *Pseudomonas aeruginosa*

**Figure 4:**
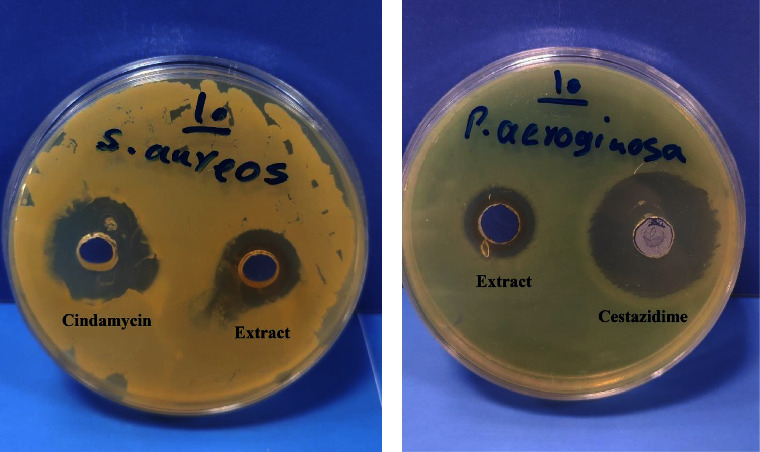
Zones of inhibitions are obtained from the extract and control solutions against (a) *Staphylococcus aureus* and (b) *Pseudomonas aeruginosa.* MIC and MBC of the extracts on *Staphylococcus aureus* and *Pseudomonas aeruginosa*.

Antibacterial activity of the extract of *Staphylococcus aureus* and *Pseudomonas aeruginosa* was investigated by the agar well diffusion susceptibility test (Figure 4).  Table 6 shows the diameters of inhibition zones obtained for different extract concentrations along with 10 *µ*g/ml clindamycin and ceftazidime solutions used as a positive control. Results of this test indicate concentration-dependent antibacterial activity of the extract against investigated microorganisms, along with higher antibacterial activity against *Staphylococcus aureus*.

Table 7 shows estimated amounts of MIC and MBC. As can be seen, MIC and MBC are the same for both microorganisms, indicating the extract's high bactericidal potency. This test's results agree with the ones obtained in the agar diffusion test and show the higher antibacterial activity of the extract against the Gram-positive*Staphylococcus aureus*.

## 4. Discussion

This study aimed to determine the effect of hydroalcoholic extract of *Scrophularia striata* on the healing of grade 1 and 2 pressure ulcers in patients with brain and spinal cord lesions. The results of daily examination of patients in terms of ulcer size during the intervention period showed significant test results in experiment group 1, experiment group 2, control group, and placebo group. Respectively, in experiment group 1, 63% of the subjects had complete ulcer healing by the tenth day of intervention. In experiment group 2, complete ulcer healing was observed for 100% of the subjects by the tenth day of the intervention. In the control group, for 27% of the subjects, complete ulcer healing occurred by the tenth day of the intervention. In none of the subjects, complete ulcer healing was observed in the placebo group until the tenth day of the intervention. Furthermore, the duration of complete ulcer healing of the patients in experiment group 2 was less than that of experiment group 1 and the control group. Also, the duration of complete ulcers healing in experiment group 2 was significantly shorter than in other three groups. Therefore, the percentage and process of ulcer healing were higher and faster in experiment group 2 than in other three groups.

The effectiveness of *Scrophularia striata* cream in ulcer healing complements the evidence of its use by native people (Ilam) and its application in traditional medicine and tissue restoration in animal studies. In this regard, the results of this study are confirmed by Shohani et al. (2010) at Ilam Medical School, by Ajdari-Zarmehri et al. (2014), in the Physiology Department of Qazvin University of Medical Sciences and Tanideh et al. (2014) in Shiraz Research Center. They studied the healing effects of *Scrophularia striata*. According to Sharifi et al., the *Scrophularia striata* plant improves episiotomy wound healing and facilitates faster healing so that it can be used to hasten episiotomy wound healing [24]. *Scrophularia striata* was also found to have antibacterial and wound-healing effects by Tanideh et al. in an animal study, indicating that the herb should be used when burn wounds are infected with *P. aeruginosa* [25]. Several antioxidant and larvicidal properties were observed for *Scrophularia striata* collected in the west of Iran [26]. *Scrophularia striata* have the potential to act as an antioxidant, but in reality, it can be concluded that the complex interaction of ecological and chemical processes which take place in the plant under such circumstances has resulted in the synthesis of different antioxidants compounds in the plant in different areas [15]. The different glycopropenoids in *Scrophularia* species reduce edema, stop cell infiltration, and have anti-inflammatory properties [20]. Phenylpropanoid glycosides inhibit the activity of macrophages, thus inhibiting the production of inflammatory chemical mediators and ultimately reducing inflammation [21].

Furthermore, the presence of phenolic acids with antibacterial properties is another reason for the effectiveness of this plant in healing skin ulcers [15]. Stimulation of fibroblast growth and antioxidant and anti-inflammatory properties of *Scrophularia striata*, along with stimulation of collagen production and faster ulcer contraction, angiogenesis, and vasodilation, confirm the effect of this plant in burn ulcer healing [16]. In specimens treated with *Scrophularia striata* extract, an increase in fibroblast cells and collagen fibres with the formation of *epidermis* and dermis was observed at the site of the ulcer [17]. Despite differences such as method, target group, and dependent variable, a double-blind, randomized controlled trial indicated that compared to chlorhexidine disinfectant mouthwash, *Scrophularia striata* decreases plaque index, pocket depth, and probing bleeding in the short term but increases the number of *Streptococcus mutans* over a long time. The findings of this study demonstrate that *Scrophularia striata* have antimicrobial properties [27].

### 4.1. Limitations and Future Directions

As a result of the COVID-19 pandemic and the limited availability of samples in this study, the study was limited in the number of samples. Furthermore, several patients were discharged from the hospital before the study time. Therefore, further studies are recommended with a larger sample size. Moreover, research about how *Scrophularia striata* cream affects open wounds caused by trauma and its effects on healing other types of ulcers, including diabetic ulcers, surgical ulcers, and burn ulcers, may be worthwhile.

## 5. Conclusion

This study showed that using *Scrophularia striata* cream accelerates the healing of pressure ulcers in first- and second-grade patients. Therefore, the use of *Scrophularia striata* is recommended to reduce the period of hospitalisation of patients following pressure ulcers and improve the quality of medical services and care.

## Figures and Tables

**Table 1:** The demographic characteristics of the patients in four study groups.

Variables		Groups				Test statistics	*P* value
		Test 1, *N* = 30	Test 2, *N* = 30	Control, *N* = 30	Placebo, *N* = 30		
		Number (%)	Number (%)	Number (%)	Number (%)		
Gender	Female	17 (56.7)	14 (46.7)	14 (46.7)	16 (53.3)	0.90^*∗*^	0.825
	Male	13 (43.3)	16 (53.3)	16 (53.3)	14 (46.7)		

Age	15–25	4 (13.3)	3 (10)	2 (6.7)	7(23.3)	10.10^*∗∗*^	0.609
	25–40	6 (20)	4 (13.3)	3 (10)	5 (16.7)		
	40–55	7 (23.3)	3 (10)	4 (13.3)	5 (16.7)		
	55–70	11 (36.7)	18 (60)	19 (63.3)	11 (36.7)		
	>70	2 (6.7)	2 (6.7)	2 (6.7)	2 (6.7)		

Marital status	Married	20 (66.7)	18 (60)	22 (73.3)	12(40)	12.94^*∗∗*^	0.121
	Single	6 (20)	9 (30)	3 (10)	13 (43.3)		
	Widow	3 (10)	1 (3.3)	3 (10)	4 (13.3)		
	Divorced	1 (3.3)	2 (6.7)	2 (6.7)	1 (3.3)		

Occupation	Employed	3 (10)	7 (23.3)	2 (6.7)	7 (23.3)	29.01^*∗∗*^	0.002
	Housewife	12 (40)	2 (6.7)	9 (30)	4 (13.3)		
	Retired	6 (20)	2 (6.7)	3 (10)	0 (0)		
	Unemployed	1 (3.3)	2 (6.7)	6 (20)	6 (20)		
	Self-employment	8 (26.7)	17 (56.7)	10 (33.3)	13 (43.3)		

Education	Illiterate	7 (23.3)	8 (26.7)	11 (26.7)	8 (26.7)	4.69^*∗*^	0.861
	Middle school	8 (26.7)	5 (16.7)	5 (16.7)	3 (10)		
	High school	8 (26.7)	8 (26.7)	6 (20)	9 (30)		
	University	7 (23.3)	9 (30)	8 (26.7)	10 (33.3)		

^*∗*^Chi-square test. ^*∗∗*^Fisher's exact test.

**Table 2:** Patient's clinical characteristics in four groups of study.

Variables	Groups	Test 1	Test 2	Control	Placebo	Test statistics	*P* value
		Number (%)	Number (%)	Number (%)	Number (%)		
Case of hospitalization	SCI	16 (53.3)	13 (43.3)	14 (46.7)	19 (63.3)	2.80^*∗*^	0.423
	CVA	14 (46.7)	17 (56.7)	16 (53.3)	11 (36.7)		

Inpatient department	ICU	21 (70)	19 (63.3)	22 (77.7)	24 (80)	11.36^*∗∗*^	0.729
	Neurosurgery	9 (30)	11 (36.7)	8 (26.7)	21 (70)		

Hospitalization	1–6	9 (30)	11 (36.7)	6 (20)	0 (0)	13.55^*∗*^	0.004
	7–12	21 (70)	19 (63.3)	24 (80)	30 (100)		

Wound type	Grade 1	4 (13.3)	7 (23.3)	6 (20)	14 (46.7)	9.87^*∗*^	0.020
	Grade 2	26 (86.7)	23 (76.7)	24 (80)	6 (53.3)		

SCI, spinal cord injury; CVA, cerebrovascular accident; ICU, intensive care unit. ^*∗*^Chi-square test. ^*∗∗*^Fisher's exact test.

**Table 3:** The results of the ANCOVA test with repeated measures comparing the mean ulcers size before and after intervention in four groups.

	Test 1, *N* = 30		Test 2, *N* = 30		Control, *N* = 30		Placebo, *N* = 30		Test statistics analyze of covariance	*P* value
	Mean ± Std		Mean ± Std		Mean ± Std		Mean ± Std			
Before intervention	1214.14	849.73	1461.54	651.57	472.32	140.91	574.93	199.61	*F* = 22.60	>0.001
The first day	1078.39	824.34	121.29	600.83	418.95	130.51	570.35	201.90	*F* = 37.23	>0.001
The second day	953.09	799.62	990.10	539.31	377.26	127.63	554.09	189.20	*F* = 52.02	>0.001
The third day	801.99	768.78	775.36	489.48	328.71	127.83	539.44	183.49	*F* = 57.13	>0.001
The fourth day	693.80	735.85	575.43	406.60	279.88	117.18	527.16	183.11	*F* = 61.38	>0.001
The fifth day	601.13	686.92	4.6.32	321.77	233.79	104.69	507.82	177.98	*F* = 59.76	>0.001
The sixth day	485.79	622.02	254.08	225.99	197.77	100.20	488.82	175.32	*F* = 57.02	>0.001
The seventh day	396.04	566.96	123.78	133.52	153.36	82.45	471.32	175.82	*F* = 55.78	>0.001
The eighth day	313.93	490.08	48.65	62.01	118.54	67.63	453.77	174.21	*F* = 58.62	>0.001
The ninth day	244.21	413.67	13.29	29.82	82.43	57.27	442.02	177.02	*F* = 61.38	>0.001
The tenth day	167.06	328.89	8.61	23.32	15.54	62.52	422.75	172.29	*F* = 70.05	>0.001
Repeated major	72.22		114.08		224.18		106.27			
*P* value	>0.001		>0.001		>0.001		>0.001			

**Table 4:** The comparison of ulcers healing of research subjects before and after intervention in four study groups.

Observation time	Wound healing	1st	2nd	3rd	4th	5th	6th	7th	8th	9th	10th	Cochran's Q test statistics	*P* value
		Number (%)	Number (%)	Number (%)	Number (%)	Number (%)	Number (%)	Number (%)	Number (%)	Number (%)	Number (%)		
Test group 1	Yes	0 (0)	0 (0)	0 (0)	0 (0)	0 (0)	4 (13.3)	5 (16.7)	7 (33.3)	13 (43.3)	19 (63.3)	110.96	>0.001
	No	30 (100)	30 (100)	30 (100)	30 (100)	30 (100)	26 (86.7)	25 (83.3)	23 (76.6)	17 (56.7)	11 (36.7)		

Test group 2	Yes	0 (0)	0 (0)	30 (100)	0 (0)	0 (0)	2 (6.7)	8 (26.7)	15 (50)	24 (80)	30 (100)	191.17	>0.001
	No	30 (100)	30 (100)	0 (0)	30 (100)	30 (100)	28 (93.3)	22 (73.3)	15 (50)	6 (30)	0 (0)		

Control group	Yes	0 (0)	0 (0)	30 (100)	0 (0)	0 (0)	0 (0)	0 (0)	0 (0)	2 (7.6)	8 (7.26)	60.69	>0.001
	No	30 (100)	30 (100)	0 (0)	30 (100)	30 (100)	30 (100)	30 (100)	30 (100)	28 (93.3)	22 (73.7)		

Placebo group	Yes	0 (0)	0 (0)	30 (100)	0 (0)	0 (0)	0 (0)	0 (0)	0 (0)	0 (0)	0 (0)	—	—
	No	30 (100)	30 (100)	0 (0)	30 (100)	30 (100)	30 (100)	30 (100)	30 (100)	30 (0)	30 (100)		

Test statistics		—	—	—	—	—	6.26^*∗∗*^	15.80^*∗∗*^	34.06^*∗*^	56.03^*∗*^	68.54^*∗*^		
*P* value		—	—	—	—	—	0.054	>0.001	>0.001	>0.001	>0.001		

^*∗*^Chi-square test. ^*∗∗*^Fisher's exact test.

**Table 5:** The comparison of the distribution of ulcers healing time in four study groups.

Mean time						Middle time				
Group	Number	Number (percentage) of events	Number (percentage) of sensor	Estimation	Standard error	95% confidence interval	Estimation	Standard error	95% confidence interval	Log-ranktest statistics	*P*value
Test group 1	30	19 (63)	11 (37)	03.9	26.0	(51.8–55.9)	10	0.44	(14.9–86.10)	88.01	>0.001
Test group 2	30	30 (100)	0 (0)	40.8	22.0	(79.7–83.8)	8	0.32	(37.7–63.8)		
Control group	30	8 (26)	22 (74)	93.9	05.0	(84.9–10.03)	—	—	—		
Placebo group	30	0 (0)	30 (100)	—	—	—	—	—	—		

**Table 6:** Results of inhibition zones determined for different concentrations of the extract.

Diameter of inhibition zone				
Microorganism	1 mg/ml extract	5 mg/ml extract	10 mg/ml extract	Control^¶^
*Staphylococcus aureus*	15.1 ± 0.2	18.1 ± 0.4	18.7 ± 0.1	25.2 ± 0.2
*Pseudomonas aeruginosa*	NF^†^	11.2 ± 0.1	16.7 ± 0.1	29.3 ± 0.1

Test 1, 1 mg/ml extract; test 2, 5 mg/ml extract; control, 10 mg/ml extract clindamycin for *Staphylococcus aureus* and ceftazidime for *Pseudomonas aeruginosa*. ^†^Inhibition zone was not formed at this concentration.

**Table 7:** MIC and MBC estimated amounts.

Microorganism	MIC	MBC
*Staphylococcus aureus*	0.3 mg/ml	0.3 mg/ml
*Pseudomonas aeruginosa*	1.25 mg/ml	1.25 mg/ml

MIC, minimum inhibitory concentration; MBC, minimum bactericidal concentration.

## Data Availability

The data used to support the findings of this study are available from the corresponding author upon request.
